# Construction of Exosomes that Overexpress CD47 and Evaluation of Their Immune Escape

**DOI:** 10.3389/fbioe.2022.936951

**Published:** 2022-06-30

**Authors:** Xin-Yu Ben, Ya-Ru Wang, Hui-Hui Zheng, De-Xian Li, Rui Ren, Pan-Li Ni, Hai-Ying Zhang, Ren-Jun Feng, Yun-Qing Li, Qi-Fu Li, Xi-Nan Yi

**Affiliations:** ^1^ Key Laboratory of Brain Science Research and Transformation in Tropical Environment of Hainan Province, Department of Neurology, The First Affiliated Hospital, Hainan Medical University, Haikou, China; ^2^ Department of Human Anatomy and Department of Neurology of the First Affiliated Hospital, Hainan Medical University, Haikou, China

**Keywords:** exosomes, cd47, immune escape, adipose stem cells, phagocytosis, macrophage

## Abstract

Our general purpose was to provide a theoretical and practical foundation for the use of exosomes (EXOs) that have high levels of CD47 as stable and efficient drug carriers. Thus, we prepared EXOs from adipose tissue-derived mesenchymal stromal cells (ADMSCs) that had high levels of CD47 (EXOs^CD47^) and control EXOs (without CD47), and then compared their immune escape *in vivo* and their resistance to phagocytosis *in vitro*. Nanoflow cytometry was used to determine the CD47 level in these EXOs, and the amount of EXOs^CD47^ that remained in rat plasma at 3 h after intraperitoneal injection. Phagocytosis of the EXOs was also determined using *in vitro* rat macrophage bone marrow (RMA-BM) experiments. Our *in vitro* results showed that macrophages ingested significantly more control EXOs than EXOs^CD47^ (*p* < 0.01), with confirmation by ultra-high-definition laser confocal microscopy. Consistently, our *in vivo* results showed that rats had 1.377-fold better retention of EXOs^CD47^ than control EXOs (*p* < 0.01). These results confirmed that these engineered EXOs^CD47^ had improved immune escape. Our results therefore verified that EXOs^CD47^ had increased immune evasion relative to control EXOs, and have potential for use as drug carriers.

## Introduction

Pan and Johnstone first described the formation and release of small extracellular vesicles by sheep reticulocytes in 1983 (Pan and Johnstone, 1983), and Johnstone et al. (1987) first used the word “exosome” to refer to these extracellular vesicles in 1987. Exosomes (EXOs) are extracellular vesicles with diameters of 30–200 nm that are secreted by diverse living cells. Many different types of eukaryotic and prokaryotic cells produce EXOs (Trams et al., 1981; Kowal et al., 2014). For example, mesenchymal stem cells (MSCs), immune cells, cancer cells, and neurons all secrete EXOs (Fauré et al., 2006; Krämer-Albers et al., 2007; Guescini et al., 2010; Lachenal et al., 2011; Lai et al., 2015). An EXO can function as a carrier of genetic material and affect many physiological and pathological functions of cells, including signal transduction pathways. In particular, EXOs can contain mRNAs, microRNAs, and proteins, and transfer these molecules to nearby or distant cells, thereby regulating the function of recipient cells (Valadi et al., 2007). Many studies showed that EXOs play important roles in nerve development, nerve regeneration, and the protection of nerve synaptic activity (Antonucci et al., 2012; Frühbeis et al., 2012; Frühbeis et al., 2013; Morel et al., 2013; Yang et al., 2015). EXOs from different sources have different proteins, but some proteins are present in all known EXOs (CD9, CD82, and ALEX), and these can be used as marker proteins to identify EXOs (Zhang and Grizzle, 2014; Chen J. et al., 2021; Xu et al., 2022). The EXOs secreted by different cells have different functions. For example, EXOs derived from MSCs can promote peripheral nerve regeneration (Mead and Tomarev, 2017), and EXOs derived from neuronal stem cells may protect the heart from ischemia/reperfusion injury by altering signaling *via* the JAK1/2 and Gp130 pathways (Katsur et al., 2021). MSCs typically produce abundant EXOs (Madrigal et al., 2014).

Because EXOs are naturally produced nano-scale carriers, they can be loaded with drugs or other substances to facilitate intercellular transport. Moreover, the lipid bimolecular membrane structure of EXOs means they can easily penetrate cell membranes, and can even pass through the blood-brain barrier. The advantages of EXOs as drug delivery carriers is that they can encapsulate a drug and other substances, such as RNase and proteases, and then deliver these substances to target cells in complete form (Alvarez-Erviti et al., 2011; Kooijmans et al., 2012). Alvarez-Erviti et al. (2011) added drugs to EXOs using electroporation and other methods to engineer EXOs for treatment of diseases. Other studies reported that EXOs can be used as drug carriers in tissues, and that EXOs are useful for damage repair and treatment of chronic diseases (Wahlgren et al., 2012; Lamichhane et al., 2015). These novel scientific and technological developments potentially have major impacts on human health and life (Chen L. et al., 2021; Wang et al., 2021), and use of EXOs as drug carriers are gradually changing the treatments for certain diseases.

Many recent studies showed that EXOs play a key role in the repair of nerve injuries (Dong et al., 2019; Salarpour et al., 2022). EXOs act on neurons, Schwann cells, microglia, and other cells of the nervous system by release of active substances that promote nerve regeneration. In 2018, Li et al. (2021) (Yin et al., 2018) described the effect of adipose-derived mesenchymal stem cell-EXOs (ADMSC-EXOs) using the sciatic nerve injury model. They reported reduced damage within the nerve bundle, a more intact and functional perineurium, reduced Schwann cell apoptosis and autophagy, and increased repair of sciatic nerve injury. In 2019, Ren Z. W. et al. (2019) developed miRNA-133b-modified ADMSC-EXOs and found that they significantly promoted the recovery of nerve function in animals with spinal cord injury due to their effects on signal pathways related to axon regeneration and the expression of NF, GAP-43, GFAP, and MBP. Engineered EXOs derived from ADMSCs can therefore be used to reduce or repair nerve damage.

Initial research of our group found that EXOs can carry miRNA-21-5p, which then acts on dorsal root ganglion neurons by downregulating the SPRY gene and protein. CD47 promotes the growth of neurites, and also down-regulates the expression of TIMP3 and reduces neuronal apoptosis. Therefore, EXOs have potential use as carriers of drugs and active substances that function in tissue repair, such as nerve regeneration. Moreover, our laboratory is currently developing certain innovations in EXO extraction technologies. In particular, we added an ultra-filtration process to the extraction of EXOs by using an “ultra-isolation” method to increase the concentration and purity of EXOs, so that they have a wider application as drug carriers.

CD47, also known as integrin-associated protein (IAP), is a transmembrane glycoprotein that is present in a variety of cells (Ren Z. W. et al., 2019). This protein has an immunoglobulin variable N-terminal domain, five transmembrane domains, and a C-terminal intracellular domain that has four different spliced isoforms (Duperrier et al., 2002). CD47 functions as the ligand of SIRPα, and their binding can inhibit phagocytosis (Chao et al., 2012). CD47 inhibits the phagocytosis and stimulates transmembrane migration of a variety of immune cells (Stefanidakis et al., 2008), and also participates in a variety of immunophysiological processes, such as inducing the apoptosis of lymphocytes (Pettersen et al., 1999) and inhibiting the release of cytokine IL-from monocytes (Avice et al., 2000). Many studies (Willingham et al., 2012) reported that tumor cells had increased expression of CD47, and this provided them with successful immune escape. Kamerkar et al. (2017) injected mice with EXOs that had a high level of CD47 or with CD47 knock-out. Their EXOs^CD47^ activated the CD47-SIRPα pathway, accumulated in the circulation, and could be used to target the oncogenic Kras protein This study inspired us to construct EXOs with the CD47 phenotype to block their phagocytosis, so they can be used to escape immune responses and function as carriers for various types of drugs. However, the EXOs^CD47^ used in previous research were derived from tumor cells, and because these EXOs could possibly induce carcinogenesis, they are not suitable for use as drug carriers. We therefore examined the development of EXOs that have potential clinical applications as drug carriers (Chundawat et al., 2021).

We previously constructed CD63-GFP lentiviral vectors, transfected them into MSCs, and extracted fluorescently labeled EXOs from the serum-free culture supernatant (Ren et al., 2017). This research also showed successful use of PKH67 to label EXOs for *in vivo* tracing, allowing evaluation of the immune escape of EXOs^CD47^. Current research on EXOs derived from MSCs is still in the exploratory stage, but animal models have demonstrated their therapeutic potential. Compared with the transplantation of stem cells, the use of appropriate EXOs is likely to have fewer adverse effects and be more suitable for tissue regeneration therapy (Caseiro et al., 2015). In the present study, we used EXOs derived from ADMSCs to construct EXOs^CD47^ as a basis for subsequent examination of their effects on neural repair. Immune escape may occur due to antigenic changes, persistent infections, or immunosuppression; the EXOs^CD47^ studied here were designed to achieve immune escape by immunosuppression.

We used ADMSCs because they are readily available, easy to grow *in vitro*, and can secrete a variety of cytokines (Heo et al., 2019; Prakoeswa et al., 2020). Recent studies showed that ADMSC-EXOs contain 148 miRNAs (Zhang et al., 2017) and 1,688 proteins, and function in a variety of cells (Ni et al., 2018). This makes ADMSC-EXOs highly suitable as carriers of therapeutic drugs.

Our general goal was to assess the feasibility of using EXOs^CD47^ for clinical applications, especially for the treatment of nerve injuries. The specific purposes of this study were to construct a CD47 overexpression lentivirus, transfect ADMSCs to obtain CD47-ADMSCs, extract and purify the EXOs secreted by these cells (EXOs^CD47^), measure the levels of CD47 in these EXOs using flow cytometry and qPCR, and examine the immune escape of these EXOs^CD47^.

## Materials and Methods

### Cell Culture

HEK293T and rat ADMSC cells were maintained in Dulbecco’s modified Eagle medium (DMEM, Hyclone, United States) that was supplemented with 10% fetal bovine serum (HyClone, United States) and 1% penicillin/streptomycin (HyClone, United States). Rat macrophage bone marrow (RMA-BM) cells were maintained in DMEM supplemented with 10% fetal bovine serum (HyClone). All cells were cultured at 37°C with 5% CO_2_ (Raessi et al., 2021).

### Plasmid Construction

CD47 was amplified using PCR with the primer sequences listed in Supplementary Table S1. Then, the amplicon was inserted into the GV492 vector (Jikai Gene) between BamHI and AgeI to create the GV492-CD47 plasmid. The final product was confirmed by sequencing, and the clone was stored at −80°C before use. GV492-CD47 was used to transfect HEK293T cells, and qPCR was used to measure the level of CD47.

### Lentivirus Package

The pHelper 1.0 vector plasmid (15 µg), pHelper 2.0 vector plasmid (10 µg), Jikai Transfection Reagent (1 ml, all from Jikai Gene), and GV492-CD47 vector plasmid (20 µg) were mixed and allowed to stand at room temperature for 15 min. The solution was dripped into the culture medium with HEK293T cells, and the culture medium was discarded after 6 h. After washing with PBS, 20 ml of complete medium was added, and the cells were grown for 72 h. After centrifugation (4,200 g, 4°C, 10 min), the supernatant was collected and passed through a 0.45-μm filter. The filtrate was transferred to an “ultra-isolation” tube (40 ml, Beckman-Coulter) and centrifuged (54,000 g, 4°C, 2 h). Then, the supernatant was removed, DMEM was added, and the sample was mixed by pipetting. After full dissolution, the sample was centrifuged again (7,800 g, 5 min), and the supernatant was aspirated. The HEK293T cells that were transfected with the CD47 lentiviral vector were examined using fluorescence microscopy and the virus titer was calculated.

### Transfection

To produce EXOs^CD47^, ADMSCs were transfected with lentiviral vectors. When the cell density was 60%–70% in 100 mm Petri dishes, the medium was supplemented with 2 ml of puromycin (6 μg/ml) as a selection medium. Then, 10 μg/ml of CD47 overexpression lentivirus (50 MOI) was added, and cells were cultivated for 1 day. The original medium was then discarded and replaced with complete medium, and the cells were cultivated to the fifth passage prior to observation of fluorescence.

### qPCR of CD47-ADMSCs

Total RNA was extracted from cells using Trizol (Invitrogen). A Nanodrop-2000 spectrophotometer (Thermo Scientific, United States) was used to measure RNA concentration. To analyze mRNA expression, 2 μg of total RNA was reverse-transcribed using the Hifair II 1st strand cDNA Synthesis SuperMix (11119ES60, Yeasen, China), and qPCR was performed using Hieff qPCR SYBR Green Master Mix (11200ES60, Yeasen, China) with the primer sequences listed in Supplementary Table S2. Each kit was used in accordance with the manufacturer’s instructions.

### Preparation of Exosomes

The detailed procedures used to prepare exosomes were described by Ren R. et al. (2019). Briefly, EXOs were extracted from the culture supernatant of ADMSCs. In this procedure, ADMSCs were washed twice with PBS, serum-free medium was added, cells were cultured at 37°C and 5% CO_2_ for 72 h, and the supernatant was then collected after centrifugation. The EXOs were then separated by ultracentrifugation. First, the sample was centrifuged (3,000 g, 15 min) to remove cell debris. Then, the supernatant was passed through a 0.22-μm filter and ultra-centrifuged at 100,000 g for 70 min. The pellet was resuspended, and the ultra-centrifugation was repeated. The resulting pellet consisted of EXOs, and was diluted in PBS and stored at −80°C until use.

### Characterization of Exosomes^CD47^

The size distribution of extracted EXOs was determined using ZetaView (Colloid Metrix, Germany), and their morphology was examined using transmission electron microscopy (JEM-2000EX TEM, Japan). The level of CD47 was determined using a nanoflow cytometer. EXOs derived from ADMSCs that were not transfected were used as a control.

### Fluorescence Tracer Quantification of Plasma Exosomes

All animal experiments were pre-approved by the local ethics committee. These experiments used healthy male Sprague-Dawley (SD) rats that were 6–8 weeks old that were provided by Changsha Tianqin Company. The two groups of rats received intraperitoneal injections with 1×10^6^ EXOs derived from control ADMSCs or transfected cells (EXOs^CD47^), and rat plasma was extracted 3 h later. The plasma sample was diluted in PBS (11 ml) and passed through a 0.2-μm filter. Nanoflow cytometry was used to analyze the positivity of PKH67-EXOs in these samples, and comparison of the two groups was used to determine the extent of immune escape.

### Western Blotting of Rat Peritoneal Lavage Fluid

At 30 min after intraperitoneal injection of EXOs, the peritoneal lavage fluid of the two groups of rats was drawn, lysed with RIPA lysis buffer at 4°C for 30 min, and the protein concentration was then measured using the Pierce BCA kit (Thermo, United States). The protein samples were concentrated on a 5% SDS-PAGE stacking gel, and then separated on a 12% SDS-PAGE resolving gel. The proteins were then transferred to a PVDF membrane. After blocking with 5% BSA, this membrane was incubated with the primary antibody at 4°C overnight, and then with the corresponding HRP-conjugated secondary antibody at room temperature for 1 h. The band was observed using an automated digital gel image developer (TANON 5200Multi, CN). The primary antibodies were rabbit anti-CD14 antibody (1:1,000, Invitrogen, PA5-95334) and rabbit anti-CD68 antibody (1:1,000, Invitrogen, PA5-78996). The secondary antibody was an HRP-conjugated goat anti-rabbit antibody (1:2000, Cell Signaling Technology, 7074P2).

### Phagocytosis of Isolated Macrophages

Immunofluorescence was used to identify RMA-BMs based on the presence of CD68. These cells were cultured in a confocal laser culture dish, and different groups of EXOs (∼1×10^8^) were added to each well, followed by incubation for 3 h. The controls consisted of PKH67-labeled non-transfected ADMSC-derived EXOs. Ultra-high-definition laser confocal microscopy (WHB Scientific, WHB-35-20-1) was used for observations at 1, 2, and 3 h after the addition of EXOs.

### Statistical Analysis

All data were expressed as means ± SEMs. Student’s *t*-test was used for comparisons of two groups, and one-way ANOVA was used for comparisons of three or more groups. A difference was considered statistically significant when the *p* value was below 0.05.

## Results

### Lentiviral Plasmid With CD47 Overexpression

**FIGURE 1:**
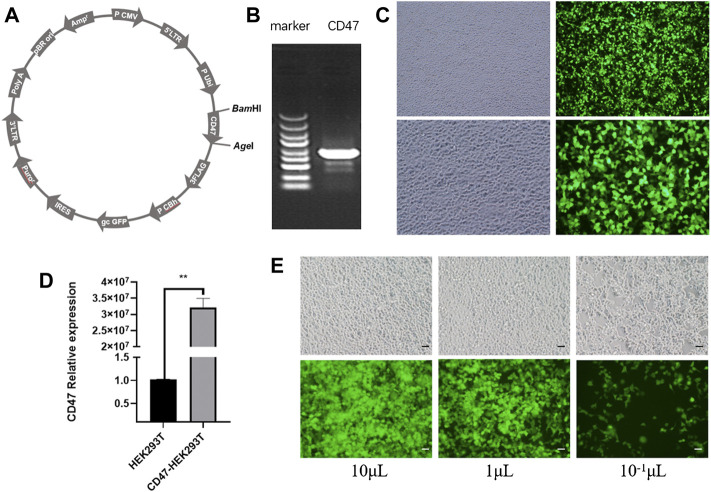
**(A)** Vector plasmid with CD47 overexpression. **(B)** Agarose gel electrophoresis of the target gene (CD47). **(C)** Phase contrast microscopy (left) and fluorescence microscopy (right) of HEK293T cells that were transfected with the CD47 overexpression plasmid. **(D)** Relative expression of CD47 (qPCR) in HEK293T cells before and after plasmid transfection. *N* = 3, **p < 0.01. **(E)** Phase contrast microscopy (top) and fluorescence microscopy (bottom) of HEK293T cells with the CD47 overexpression lentiviral vector after transfection. Scale bar: 500 μm.

We constructed a GV492-CD47-GFP recombinant plasmid as the first step in engineering EXOs that overexpress CD47 from *Rattus norvegicus* (Figure 1A). Agarose gel electrophoresis confirmed that CD47 had a size of about 950 bp (Figure 1B). We then transfected HEK293T cells with these CD47 overexpression plasmids. The results showed green fluorescence, indicating successful overexpression of CD47 (Figure 1C), and significantly greater expression of CD47 in transfected cells than control cells (Figure 1D). To ensure the CD47 overexpression plasmid was stably expressed during the passage process, we used lentivirus to wrap the CD47 overexpression plasmid, and then transfected the HEK293T cells again. The results indicated green fluorescence (Figure 1E), and measurements of transduction units (TU) indicated about 1 × 10^9^ TU/ml.

### Development and Characterization of Exosomes^CD47^

**FIGURE 2:**
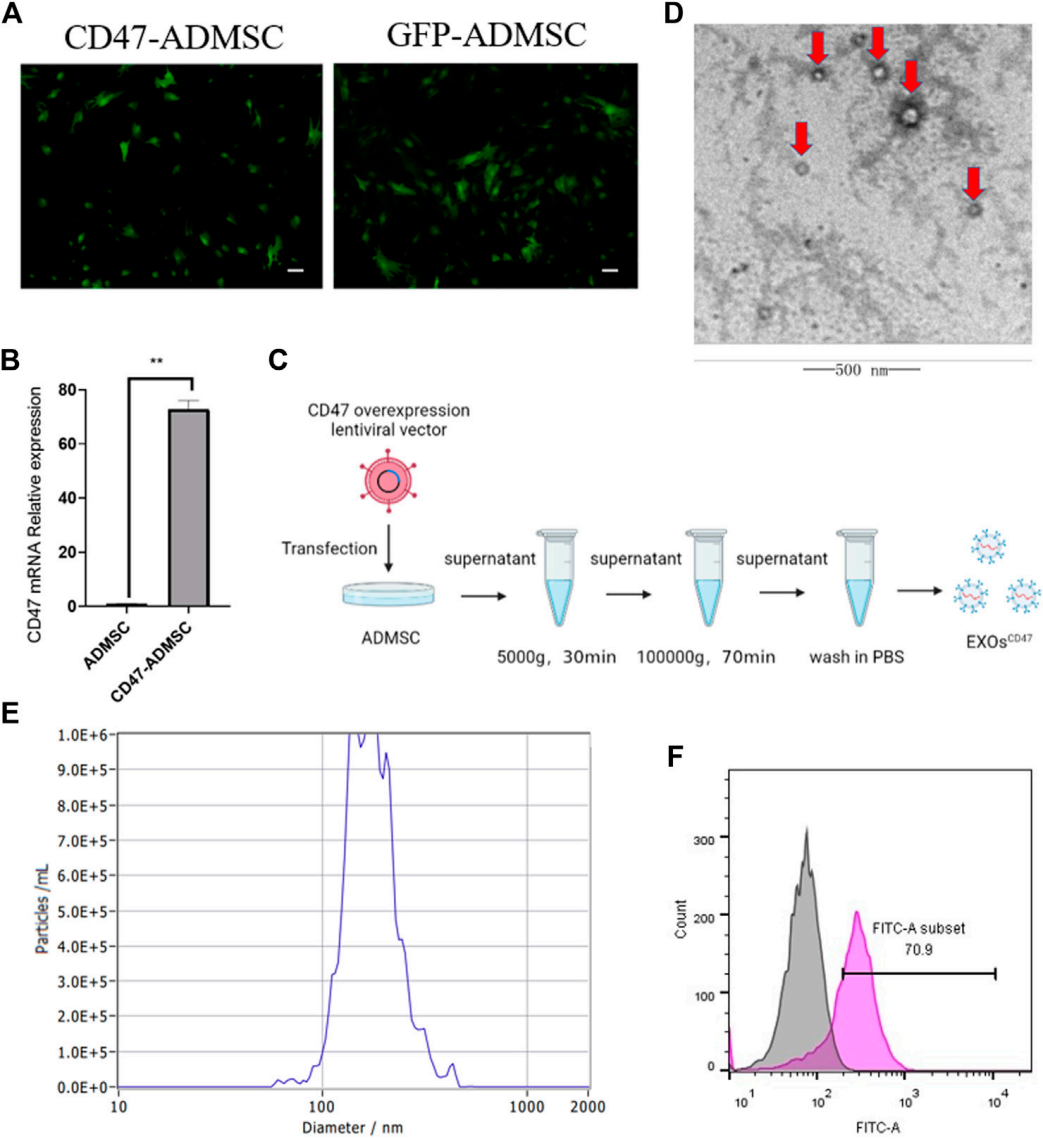
**(A)** Fluorescence microscopy of ADMSCs with the CD47 overexpression lentiviral vector at the fifth passage after transfection and GFP-ADMSCs (positive control). Scale bar: 500 μm. **(B)** Relative expression (qPCR) of CD47 in ADMSCs and CD47-ADMSCs at the fifth passage after transfection. N = 3, **p<0.01. **(C)** Experimental procedure used to extract EXOs. **(D)** Electron microscopy of EXOs produced by ADMSCs (red arrows). Scale bar: 500 nm **(E)** Size distribution of EXOs produced by ADMSCs. **(F)** Nanoflow cytometry of CD47 expression by EXOs^CD47^. Pink: EXOs^CD47^; Grey: ADMSC-EXOs.

To obtain EXOs with high levels of CD47 (EXOs^CD47^), we transfected ADMSCs with a lentiviral vector containing a CD47 overexpression plasmid, conducted screening using puromycin, and then examined cells from the fifth generation (Figure 2A). The results indicated higher expression of CD47 in fifth-generation CD47-ADMSCs than in control fifth-generation ADMSCs (Figure 2B). We then extracted EXOs from these cells using ultrafiltration and “ultra-isolation” (Figure 2C). Transmission electron microscopy showed that the vesicles secreted by the transfected ADMSCs had morphological characteristics that were typical of EXOs (Figure 2D). Nanoparticle analysis showed that the particle size distribution of these EXOs was within the normal range for EXOs (Figure 2E). We then used nanoflow cytometry to determine the CD47 level in the EXOs secreted by CD47-ADMSCs relative to fifth-generation controls. The results indicated the level of CD47 in EXOs^CD47^ was about 1.7-times greater than in ADMSC-EXOs (Figure 2F).

### Immune Escape of Exosomes^CD47^ in Rats

**FIGURE 3:**
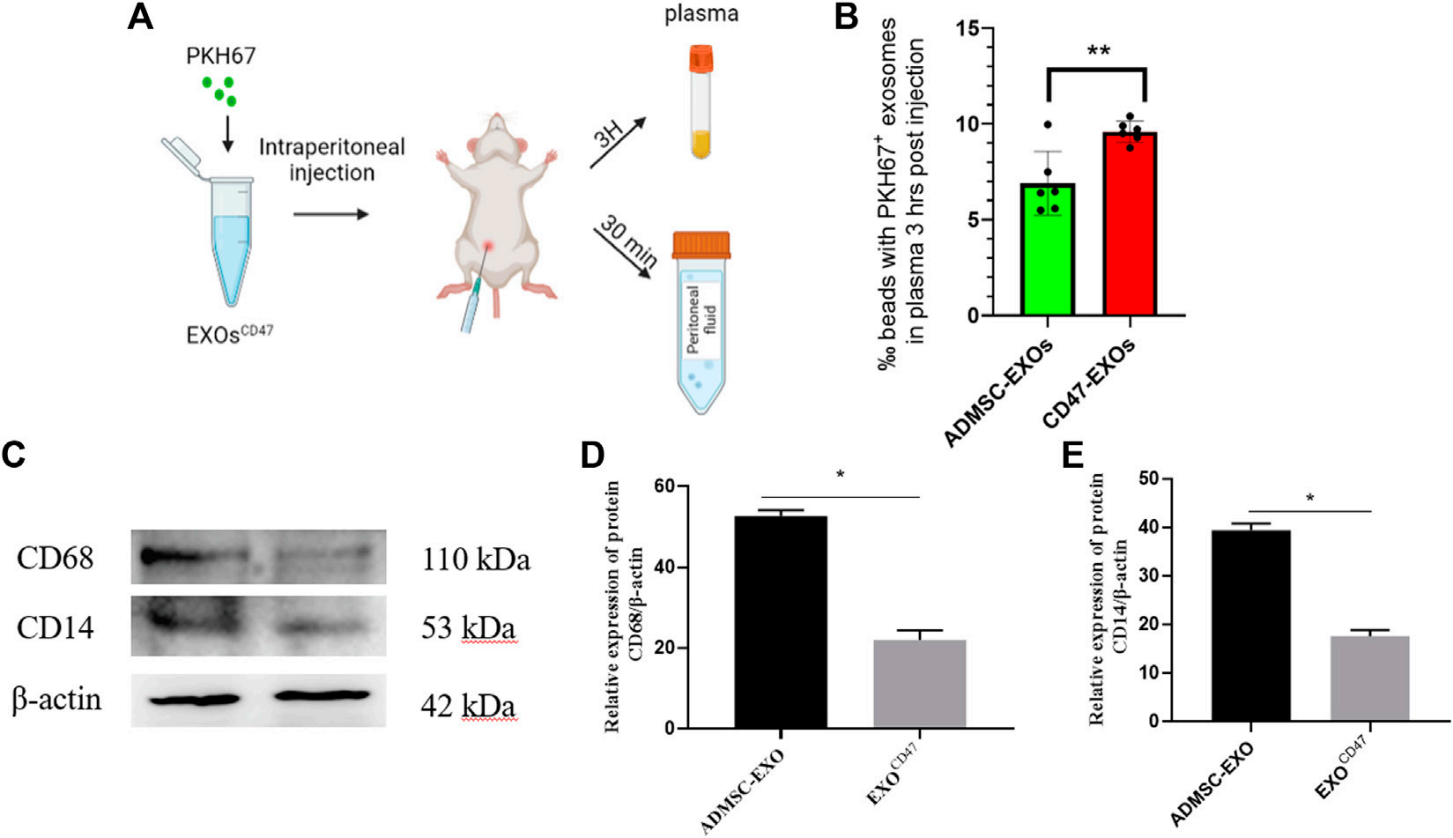
**(A)** Experimental procedure used to examine the *in vivo* stability of EXOs. **(B)** Retention of EXOs at 3 h after intraperitoneal injection of ADMSC-EXOs (control) and EXOs^CD47^. *n* = 6, **p<0.01. **(C–E)** Western blotting of lavage fluid for CD68 and CD14 (macrophage markers) in the ADMSC-EXOs (control) and EXOs^CD47^ groups. *N* = 3, *p < 0.05.

To test the immune escape of EXOs^CD47^ *in vivo*, we prepared PKH67-labeled EXOs^CD47^ and ADMSC-EXOs (control) and administered them by intraperitoneal injection into rats (1 ml containing 1×10^6^ EXOs, six rats per group, Figure 3A). After 3 h, rat plasma was extracted, and total plasma EXOs were extracted by ultrafiltration and “ultra-isolation”. Nanoflow cytometry showed greater plasma retention of EXOs^CD47^ than ADMSC-EXOs (Figure 3B), suggesting increased immune escape by the EXOs^CD47^.

To determine whether the PKH67-labeled exosomes triggered the phagocytosis of macrophages in the abdominal cavity, we collected peritoneal lavage fluid from rats at 30 min after injection of the PKH67-labeled exosomes. Western blotting showed that two macrophage markers (CD14 and CD68) were significantly down-regulated in the EXOs^CD47^ group (Figure 3C). These results suggest that EXOs^CD47^ are less likely to induce macrophage phagocytosis in the abdominal cavity (Figure 3C).

### Immune Escape of Exosomes^CD47^ *in vitro*

**FIGURE 4:**
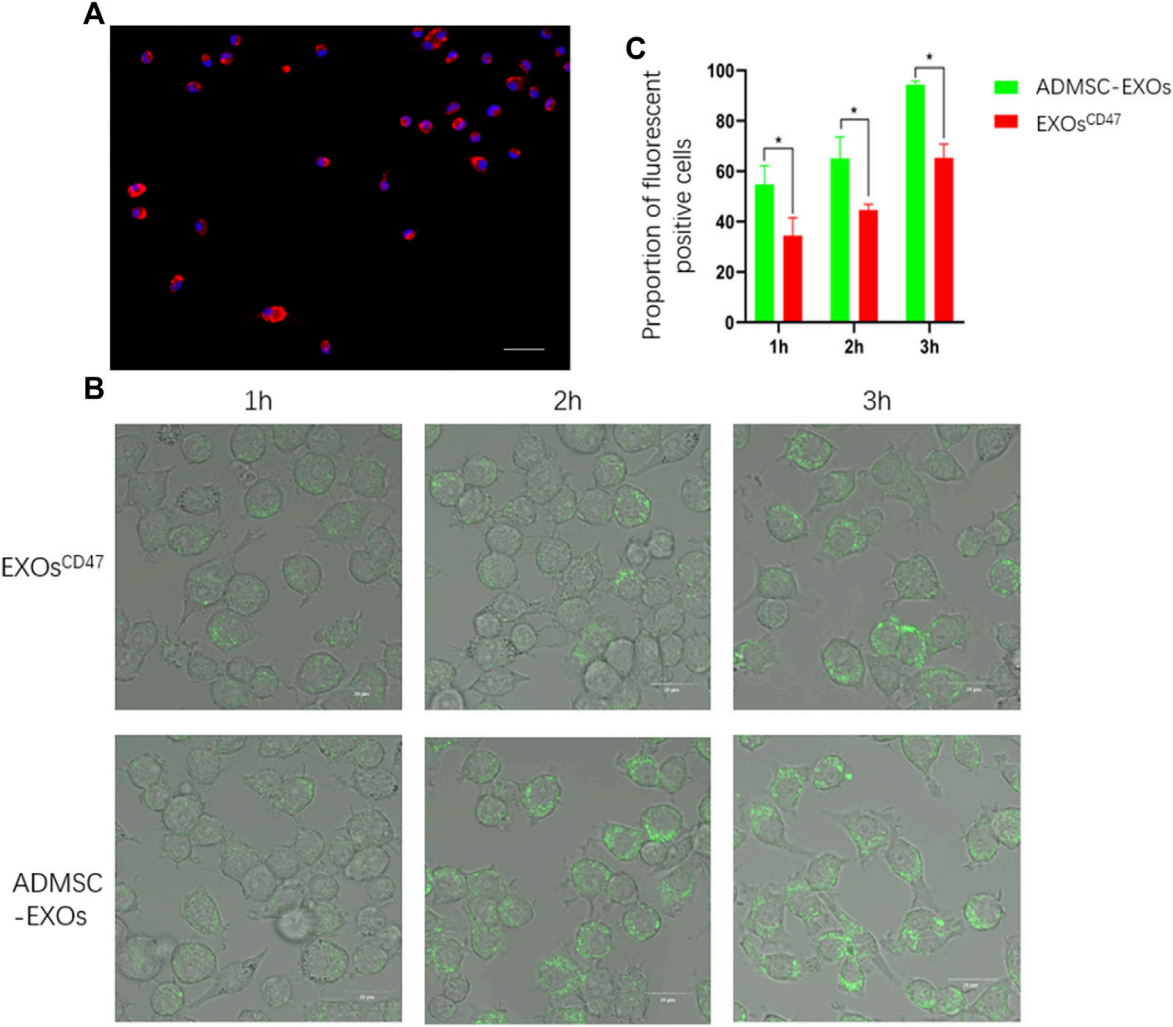
**(A)** Immunofluorescence analysis of isolated macrophages. Blue: DAPI, Red: CD68, scale bar: 100 μm. **(B)** Representative ultra-high-definition laser confocal microscopy of the phagocytosis of EXOs from the ADMSC-EXOs (control) and EXOs^CD47^ groups after 1, 2, and 3 h scale bar: 20 μm. **(C)**. Quantitation of the results in Figure 4B. *p < 0.05, *n* = 3.

We performed immunofluorescence microscopy of RMA-BM to confirm they had the general characteristics of macrophages (Figure 4A). To assess immune escape of EXOs^CD47^ *in vitro*, we added PKH67-labeled EXOs^CD47^ or control EXOs into RMA-BM cell culture medium, and then analyzed these cells using ultra-high-definition laser confocal microscopy after 1, 2, and 3 h. The results indicated significantly greater engulfment of ADMSC-EXOs than EXOs^CD47^ (Figures 4B,C).

## Discussion

Our *in vivo* and *in vitro* experiments indicated successful construction of functionalized EXOs derived from ADMSCs that had high levels of CD47 and were able to escape phagocytosis by macrophages. Many recent studies have used nanoparticles in diverse medical applications, most notably for experimental treatment of cancer (Roostaee and Sheikhshoaie, 2020; Chan et al., 2022; Roostaee et al., 2022). These EXOs^CD47^ thus have potential for use in subsequent research as drug carriers.

Previous studies found that CD47, a transmembrane glycoprotein, is present in a variety of cells, that its expression is significantly increased in tumor cells (Hu et al., 2021), and there is also some increased expression in normal ADMSCs. Other studies showed that the EXOs secreted by some tumor cells have roles in the onset and development of tumors (Whiteside, 2016). Although tumor cells produce EXOs with high levels of CD47, they are obviously not suitable for use as drug carriers in clinical practice. Instead, we chose ADMSCs as the source of EXOs^CD47^ because previous studies of the EXOs secreted by these cells reported no adverse reactions (Kibria et al., 2016).

A recent study reported that the CD47 level in EXOs from the plasma of breast cancer patients was higher than in healthy controls (Hu et al., 2021). Another study reported that the retention of EXOs derived from fibroblasts of mice with CD47 knockout was reduced *in vivo* (Kamerkar et al., 2017). This led us to hypothesize that a cell line with overexpression of CD47 can produce EXOs^CD47^ that have greater immune escape function. Our design of the CD47 overexpression plasmid was based a previous study (Ye et al., 2014). Thus, we added a lentivirus to the plasmid so that CD47 was stably expressed during cell passage. We then identified EXOs^CD47^ using nano-flow cytometry, which has certain advantages over western blotting for CD47, as employed by Du et al. (2021).

In this study, we performed intraperitoneal injection of EXOs into rats, as described by Kamerkar et al. (2017). We used this method because peritoneal washing fluid can be easily collected and analyzed to determine the chemotaxis and aggregation of macrophages. Administration of EXOs by other methods does not have this advantage. Kamerkar et al. (2017) extracted plasma from orbital blood to determine the retention of plasma EXOs, and then used a nanoparticle analyzer to measure the concentration and number of retained EXOs. We collected blood by perfusion, extracted plasma, and then performed nanoflow cytometry to detect the retention of EXOs.

To further verify the anti-macrophage effect of EXOs^CD47^, we designed an *in vitro* macrophage phagocytosis experiment to examine the relationship between EXOs and macrophages. The results showed that macrophages ingested significantly more control EXOs than EXOs^CD47^ at 1, 2, and 3 h. These results indicate that the EXOs^CD47^ described here had resistance to ingestion by macrophages, although they were ultimately phagocytized. This result supports our *in vivo* results regarding the prolonged residence time of EXOs^CD47^ in the blood. We used high-resolution confocal laser microscopy in these experiments to observe the phagocytosis of macrophages, and the results provided more definitive evidence of the anti-phagocytosis activity of EXOs^CD47^.

## Conclusion

Many previous studies showed that EXOs have potential for use as agents that deliver therapeutic drugs, especially in the nervous system because EXOs may allow specific drugs to pass through the blood-brain barrier (Tian et al., 2018). However, the therapeutic potential of EXOs is limited due to their phagocytosis by macrophages (Yong et al., 2017). Therefore, we used ADMSCs to construct EXOs^CD47^. These EXOs^CD47^ have tissue repair function and increased immune escape, increasing their potential to function as drug carriers. In the future, we plan to examine the effects of loading these EXOs^CD47^ with drugs that repair nerve damage. We also plan to examine the possibility of mass production of EXOs^CD47^ to provide a theoretical and practical foundation for their use in commercial and clinical applications.

## Data Availability

The original contributions presented in the study are included in the article/Supplementary Material, further inquiries can be directed to the corresponding author.
