# Supplementary material for: Construction of Exosomes that Overexpress CD47 and Evaluation of Their Immune Escape
Source: Front Bioeng Biotechnol. 2022 Jun 30;10:936951. doi: 10.3389/fbioe.2022.936951 (PMC9279928; doi:10.3389/fbioe.2022.936951)
Supplement: Supplementary file 1 [file DataSheet1.docx]

**Table S1. CD47 gene amplification primers.**

| **ID** | **Sequence** |
| --- | --- |
| CD47-p1 | 5’-AGGTCGACTCTAGAGGATCCCGCCACCATGTGGCCCTTGGCGGCGGC-3’ |
| CD47-p2 | 5’-TCCTTGTAGTCCATACCGTTATTCCTAGGAGGTTGTATAG-3’ |

**Table S2. Primers used for RT-qPCR.**

| CD47-Forward | 5’-GTGACAGATGTGCAGGGACA-3’ |
| --- | --- |
| CD47-Reverse | 5’-AGGCACACACATAACCTCCG-3’ |
